# Expired Patents

**Published:** 1907-11

**Authors:** 


EXPIRED PATENTS.

Oct. 14, 1907.

438566. Dental Tool, F. T. Van Woert, Brooklyn, N. Y.

Oct. 28, 1907.

439238. Electro Dental Heater, L. A. Faught, Philadelphia, Pa.
439305. Method of Preparing Artificial Gums for Dental Work, Datus E. Rugg, New York, N. Y.
439427. Dental Tool Holder, C. M. Richmond, New York, N. Y.
11118. Reissue, Dentist's Burring Tool, A. W. Brown, Prince's Bay, N. Y.

The above list of patents are furnished by Davis & Davis, Solicitors of American and Foreign Patents, Washington, D. C. and St. Paul Building, New York City.

